# Intestinal organoid models as tools to interrogate the physiology of human mucosal tissues and host-microbe interactions

**DOI:** 10.1128/msphere.00820-24

**Published:** 2025-08-07

**Authors:** J. M. Lemme-Dumit

**Affiliations:** 1 Department of Pediatrics, Center for Vaccine Development and Global Health, University of Maryland School of Medicine 12264, Baltimore, Maryland, USA; 2 Department of Microbiology and Immunology, University of Maryland School of Medicine 12264, Baltimore, Maryland, USA; Virginia-Maryland College of Veterinary Medicine, Blacksburg, Virginia, USA

**Keywords:** human intestinal model, organoids, intestinal physiology, host response, multi-omics, translational research

## Abstract

The intestinal epithelium serves as a critical interface between the external environment and internal tissues, coordinating nutrient absorption, immune defense, and barrier integrity. Discerning the processes that maintain gut homeostasis has been challenging due to the complexity of the intestinal microenvironment and the difficulty in accessing human tissue. The advent of human intestinal organoid technology has transformed the field by providing relevant *in vitro* models that recapitulate the cellular diversity and function of the gut epithelium. A recent advance involves the integration of immune cells into organoid cultures, enabling the study of epithelial-immune cell interactions in both health and disease. Furthermore, the application of cutting-edge multi-omics approaches, including transcriptomics, proteomics, and metabolomics, has enabled a deeper understanding of intestinal cell signaling, niche factors, and host-microbe dynamics. These innovations have led to breakthroughs in translational research, particularly in the field of precision medicine. This minireview highlights how intestinal organoids derived from human tissue stem cells, coupled with high-resolution omics technologies, are advancing our knowledge of intestinal physiology, host responses, and disease mechanisms. It also describes the emergence of patient-derived organoids as tools to guide personalized therapeutic strategies for conditions such as inflammatory bowel disease and cystic fibrosis. As organoid models continue to evolve, the integration of additional tissue components—such as diverse immune cell lineages, stromal elements, vasculature, neural cells, and microbiota—will more accurately replicate the intricate nature of human physiology and broaden their translational potential.

## INTRODUCTION

A single-cell layer of epithelial cells lines the intestinal mucosal surface and creates an integral barrier between the external environment and internal tissues. It regulates nutrient absorption, fluid balance, and host defense. This epithelial barrier is composed of stem cells, transit-amplifying cells, absorptive enterocytes, and specialized epithelial cells, including mucus-producing goblet cells, antimicrobial peptide-producing Paneth cells, hormone-producing enteroendocrine cells, and chemosensory tuft cells (1). Tight junction proteins connect adjacent epithelial cells and maintain barrier integrity, while an interactive communication between the epithelium and underlying immune cells supports homeostasis and defense against dangerous cues (2).

Innate and adaptive immune cells interact closely with the epithelial cell layer through intercellular signals that regulate inflammation, tissue repair, and epithelial barrier integrity (2, 3). Understanding the intricate interactions between the epithelium and underlying immune cells in humans is essential for deciphering the mechanistic aspects of intestinal mucosal defense and for the development of effective therapeutics. Dysregulation of these cell-to-cell interactions can lead to a variety of disorders, including inflammatory bowel diseases, infections, metaplasia, and cancer (4, 5). Dissecting the cross-communication of epithelial-immune cells in humans has been challenging due to the complexity of the intestinal microenvironment and limitations in accessibility to human tissue.

Human intestinal organoids have revolutionized epithelial cell biology (6). These three-dimensional (3D) tissue stem-cell-derived cultures recapitulate the cellular composition and functionality of the intestinal epithelium, providing a physiologically relevant culture system for mechanistic interrogations (7–9). Efforts have been made to incorporate immune cells into intestinal tissue-derived organoids, seeking to recreate the epithelial-immune cell dynamics that occur *in vivo* and participate in gut homeostasis and inflammation (10–13). The epithelial-immune cell co-culture approach enables detailed exploration of the molecular signals that guide epithelial cell fate, mucosal barrier function, and host defenses.

Advanced technical approaches, including single-cell and high-resolution spatial transcriptomics, proteomics, metabolomics, genomics, and epigenomics, have facilitated mechanistic interrogations of tissue biology. When integrated with human intestinal organoid models, these technologies enable unprecedented exploration of cellular and molecular mechanisms with high resolution, furthering the identification of novel signaling pathways, mapping of receptor-ligand interactions, characterization of cell-to-cell communication networks, and tracking of cell fate decisions over time. These approaches are making breakthroughs in translational research, particularly in key areas such as drug discovery, infectious diseases, and the development of precision and regenerative medicine.

In this minireview, I highlight how the use of cutting-edge omics approaches applied to intestinal organoid studies has advanced our understanding of gut physiology and host-microbe interaction. Mucosal defenses against various insults are deployed by coordinated communications and adaptations among intestinal epithelial cells, immune cells, and targeted molecular mediators. Focusing on research using human tissue stem-cell-derived intestinal organoids, I will discuss the impact of omics technologies in elucidating intestinal disorders and the potential use of patient-derived intestinal organoids to identify therapeutic strategies and effective treatments. This review does not cover studies involving organoids derived from embryonic or induced pluripotent stem cells, as these topics have been reviewed elsewhere (6, 14, 15).

## HUMAN INTESTINAL ORGANOIDS

**Fig 1:**
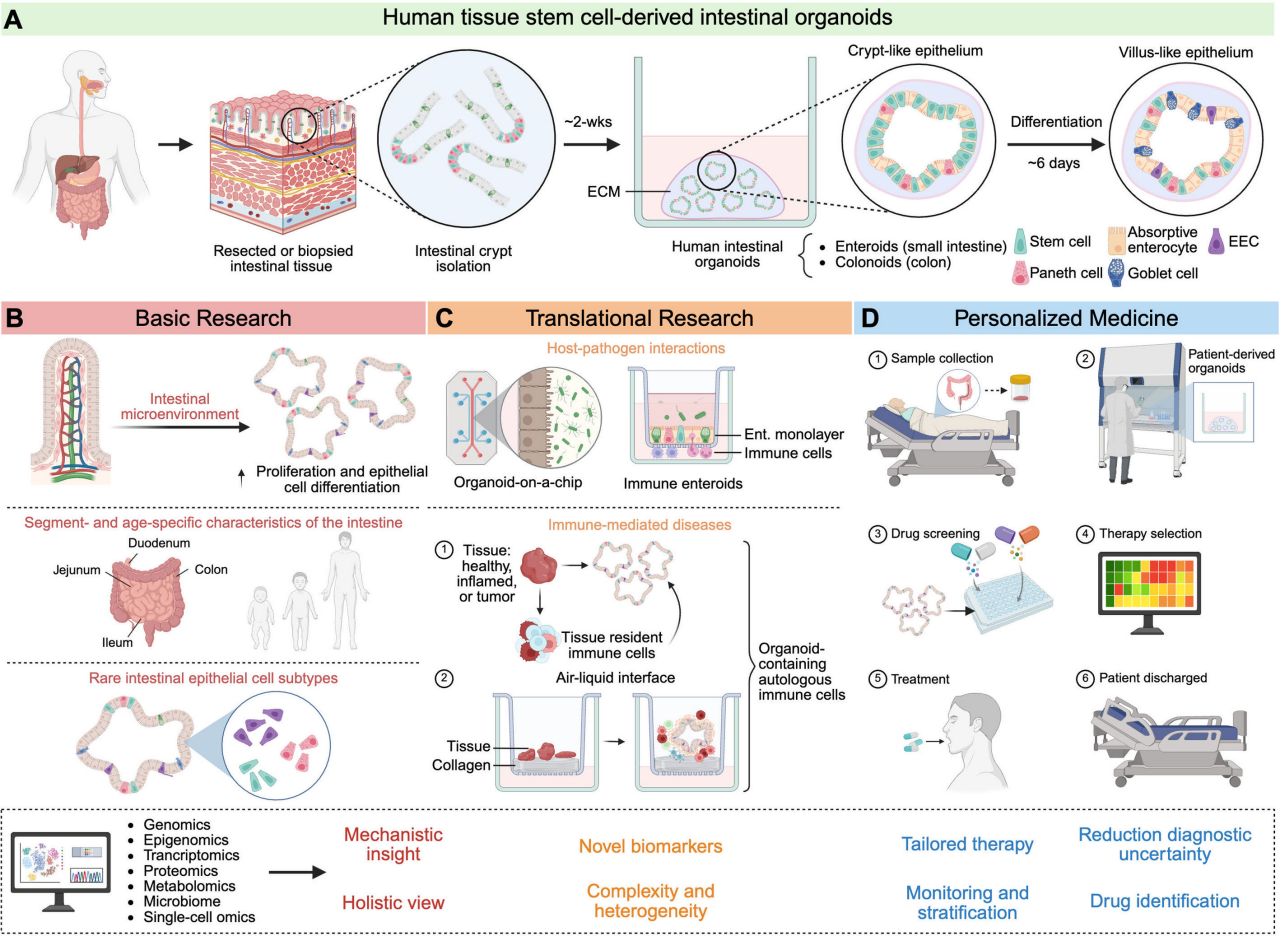
Human intestinal organoids and applications. (**A**) Intestinal crypts containing stem cells are isolated from resected or biopsied tissue and embedded in ECM along with organoid culture media. These cultures develop into enteroids or colonoids, depending on the intestinal segment, forming 3D cystic structures with the luminal compartment inward. The epithelial layer of these structures is typically enriched with stem cells and Paneth cells, closely resembling a crypt-like epithelium. Differentiation of enteroids and colonoids is induced by withdrawing key stem cell-supporting signals (i.e., Wnt and R-spondin1) from the culture media. (**B**) Research has focused on characterizing the intestinal niche microenvironment to enhance *in vitro* expansion and differentiation of intestinal organoids. Because human intestinal organoids preserve age- and segment-specific features of the donor tissue, they have been utilized to study the physiology of rare epithelial cell populations. (**C**) Human intestinal organoids are useful to study host-pathogen interactions using various platforms, such as gut-on-a-chip systems or semipermeable tissue culture inserts. The incorporation of immune cells further increases the complexity of these models. Organoids co-cultured with autologous immune cells facilitate the investigation of immune-mediated diseases and tumor biology. (**D**) Patient-derived organoids enable the evaluation of personalized and effective therapeutics. The integration of human intestinal organoids with multi-omics approaches empowers research from basic discovery to clinical applications. Figure created in BioRender (J. Lemme, 2025, https://BioRender.com/6mz0ng3).

Tissue stem-cell-derived organoids from the human small intestine and colon (known as enteroids and colonoids, respectively) have proven to be valuable *in vitro* models to study intestinal physiology (16, 17). Unlike primary cells, which exhibit a limited replication capacity, organoids can expand through serial passaging for years under optimal culture conditions due to stem cell self-renewal (18). Intestinal crypts from healthy or diseased tissues containing stem cells are cultured in solubilized extracellular matrix (ECM) supplemented with growth factors that support continuous stem cell proliferation (Fig. 1A) (18). The removal of proliferative growth factors from the culture media arrests stemness while inducing epithelial cell differentiation (Fig. 1A) (19). Notably, frozen human intestinal organoids can be cultured at any stage of the expansion process, facilitating the establishment of biorepositories (20, 21). Detailed protocols for the generation of organoids from cryopreserved tissues are widely accessible (22–24). Organoids derived from frozen specimens maintain tissue features similar to those of organoids produced from fresh samples, and their use simplifies and adds flexibility to the experimental planning and execution (23). However, a more thorough characterization of the precise effects of freeze-thaw cycles on cell genetics and phenotype is needed to ensure the reliability of cryopreserved organoids for translational studies.

### Identification of niche factors that support intestinal stem cell maintenance

Single-cell genomic techniques provide unparalleled resolution for identifying cell types, states, and overall composition within a sample (25). This approach has uncovered multiple tissue-specific signals that maintain stem cell proliferation (Fig. 1B). One study in particular identified niche factors that support human intestinal stem cells and preserve the cellular heterogeneity of the intestinal epithelium (26). Through pairwise gene expression analysis of the receptor tyrosine kinases (RTKs) in human colonic organoids and RTK ligands produced by human intestinal subepithelial stromal cells, Fujii et al. identified insulin-like growth factor 1 and fibroblast growth factor 2 as promoters of stem cell self-renewal and multi-lineage differentiation (26). More recently, Yang and colleagues reported a refinement in the composition of human intestinal organoid media that balances self-renewal and differentiation within the same organoid structure (27). This was achieved by supplementing the culture medium with three small molecules: trichostatin A (a histone deacetylase inhibitor), 2-phospho-L-ascorbic acid (vitamin C), and CP673451 (a platelet-derived growth factor receptor inhibitor) (27). Thus, the manipulation of niche factors to accurately capture intestinal epithelial cell heterogeneity represents a valuable strategy for generating physiologically representative *in vitro* systems. However, *in vivo* cell specialization within the epithelium is determined by gradients of growth factors and molecules with distinct separation between the crypt (the proliferative region) and the villus (the more differentiated region). Hence, the enrichment of culture media with specific factors remains an approximation of the natural tissue environment. Other signaling pathways affecting the regenerative capacity of stem cells have been identified. For instance, a study in mice demonstrated that retinoic acid regulates intestinal homeostasis and cell differentiation (28). Image-based screening and gene analysis of murine small intestine organoids reported that a retinoid X receptor (RXR)-α inhibitor halts absorptive enterocyte differentiation while promoting stem cell renewal (29). Future investigations using human organoids derived from diseased tissue (e.g., ulcerative colitis) could examine the therapeutic potential of RXR antagonist, promoting intestinal wound healing. The identification of key niche factors that support intestinal stem cell survival and *in vitro* proliferation provides compelling evidence for their capacity for long-term self-renewal. As more high-resolution data become available, establishing standardized culture media and protocols for tissue-specific applications will be essential for ensuring reproducibility and proper comparisons of research outcomes across studies.

### Age- and segment-specific function

Enteroids and colonoids can be generated from any segment of the small or large intestine, and they retain the phenotype and characteristics of the donor tissue from which they originated (Fig. 1B) (17, 30). Studies on enteroids derived from the pediatric duodenum and jejunum revealed morphological, functional, and genetic differences compared to their adult counterparts (31, 32). Shorter epithelial cell height and increased epithelial permeability in pediatric enteroids reflect an early-life developmental stage (31, 32). In contrast to enteroids derived from adult tissue, pediatric enteroids exhibit distinct transcriptional signatures associated with nutrient absorption, bile acid transport, and lipid metabolism, indicative of increased functionality and effective adaptations to support rapid growth and high nutritional demands (32). These observations highlight the fact that human intestinal organoids accurately model age-related features, making them valuable for studying age-specific disorders and potential treatments.

One of the major functions of the intestine is the absorption of nutrients, including glucose, amino acids, fatty acids, vitamins, and minerals, into the bloodstream and lymphatic system. Transcriptomic analysis of human intestinal segments (e.g., ileum, colon, and rectum) revealed segment-specific expression patterns of solute carrier (SLC) transporters, indicating that the same cell type (i.e., absorptive enterocyte) exhibits segment-specific functions along the intestinal tract (33). Similarly, human intestinal organoids feature segment-specific transporters; SLC transporters for bile salts, vitamins, sugars, and various amino acids are abundantly expressed in ileal-derived organoids, whereas transporters for neutral amino acids, choline, anion exchangers, glucose, and nucleic acid components are predominantly expressed in large intestinal organoids (33). Thus, gene expression profiling of human intestine revealed a complex, segment-specific organization optimized for nutrient absorption, which is preserved in organoids.

Similarly, the regional distribution of pattern recognition receptors along the gastrointestinal tract is conserved in organoid cultures (34). These innate immune pathways are preprogrammed within tissue-resident stem cells primarily during development rather than by microbial exposure (35).

### Investigating rare intestinal cell populations and their role in physiology

Methods for differentiation of intestinal tissue organoids have made possible the expression and investigation of rare cell populations, such as enteroendocrine (EEC) and enterochromaffin cells present in the human intestine (Fig. 1B). Transcriptomic and proteomic methods have uncovered features in EEC-enriched human organoids that are different from those reported for EEC in mice (36). Importantly, gene expression analyses revealed similarities between EEC in intestinal organoids and human biopsies, including the expression of markers associated with energy homeostasis (e.g., FGF21, KLB, and FGF14), neurotransmission (e.g., TAC3, FGF14), metabolic enzymes (e.g., CPB1, TDO2), and stress response (e.g., MDK, C10ORF10, and LCN15) (36). A hallmark of the EEC is its ability to produce and secrete hormones and other signaling molecules in response to stimuli. These molecules target specific cells, triggering changes in cell activity, ultimately regulating various physiological processes, such as glucose homeostasis, appetite regulation, gastrointestinal motility, and immune responses (37). EEC-enriched organoids produce a broad range of hormones, including glucagon-like peptide-1 (a glucose regulator), ghrelin (an appetite-inducing hormone), and motilin (a regulator of gut contractions), among others, suggesting that multiple subtypes of EECs can be expressed in human organoids (36). Similarly, studies in organoids have shown that the short-chain fatty acid butyrate (a metabolite produced by commensal microorganisms) limits the expansion of tuft cells—another rare type of epithelial cell that senses the intestinal environment and elicits type 2 immunity against helminths (38).

A distinct subtype of mature absorptive cells, characterized by BEST4 expression, has been identified by single-cell RNA sequencing (scRNA-seq). Although BEST4^+^ cells are relatively rare (<5% of all epithelial cells), they are consistently present throughout all regions of both the small and large intestine (39). Their differentiation depends on Notch signaling and the transcription factor SPIB (39, 40). Microscopy images have shown that BEST4^+^ cells are located adjacent to goblet cells, which express Notch ligand, suggesting cell-to-cell communication (40). Functionally, BEST4 cells express the cystic fibrosis transmembrane conductance regulator (CFTR), implicating them in the regulation of electrolyte and fluid homeostasis (40). Strikingly, IFN-γ promotes the expansion of BEST4^+^ cells, indicating a potential role in type I immune responses (39, 40).

Microfold (M) cells are a relatively rare subset of epithelial cells located within the follicle-associated epithelium that overlies Peyer’s patches (41). These specialized cells are uniquely adapted to sample luminal antigens to underlying immune cells, participating in the initiation of mucosal immune responses (42, 43). Insights into the molecular mechanisms that drive M cell differentiation have been gained through studies using murine intestinal organoids (44). A key signaling pathway involves the receptor activator of nuclear factor kappa-B, which, upon engagement, activates the NF-κB pathway through the adaptor protein TRAF6 (44). This signaling cascade is essential for the commitment of epithelial progenitors to the M cell lineage. Activation of NF-κB not only induces the expression of the transcription factor SPIB (45) but also promotes the functional maturation of the cell (44).

Collectively, these observations exemplify the utility of organoid models in investigating the functional responses of minor epithelial cell types and in elucidating the molecular mechanisms that regulate their differentiation and maturation.

### Enteroid monolayer configuration

3D human intestinal organoid cultures in ECM can be adapted to grow as monolayers on ECM-coated, semipermeable membrane tissue culture inserts. This design enables concurrent access to both the apical (luminal) and basolateral (serosal) epithelial cell membranes, thereby expanding options for experimental applications and sample collection (8, 10, 11, 17, 46, 47). For instance, human intestinal monolayers have been used to investigate epithelial cell responses to human milk, bacterial species of the human gut microbiota, and to screen probiotic bacteria through controlled apical exposure (31, 48, 49). Numerous reports in the literature have utilized enteroid monolayers to study the pathophysiology of enteric organisms, particularly those that are human-restricted, as described below. Immune cells have been incorporated into enteroid and organoid cultures to enhance their biological relevance (discussed in detail below). These co-cultures, encompassing key cellular components, are useful to understand tissue physiology, host-microbe interactions, and tissue responses in normal and pathological states.

### Bioengineered platforms

When integrated into microfluidic platforms (i.e., gut-on-a-chip), intestinal organoids benefit from dynamic elements such as controlled fluid flow, nutrient exchange, and mechanical cues, providing a better representation of the physical forces present in the native tissue environment (Fig. 1C). The chip contains a porous membrane that divides a central channel to establish two independent chambers (i.e., luminal and serosal), which are seeded with viable dissociated organoids and medium (50). The perfusable central channel system supports their growth and removes shed apoptotic cells, thereby extending tissue lifespan (51). Gut-on-a-chip models have also incorporated human intestinal microvascular endothelial cells (HIMECs) (51). The epithelial monolayer folds in response to media flow and membrane stretch, appearing to form crypt- and villus-like structures. Although proliferative cells localize in the crypt-like domains, the tissue structure lacks clear organization, failing to recreate the intestinal crypts and subepithelial compartments in the villus-like protrusions. Other bioengineered platforms have been developed to model the tissue architecture of the small intestine (including crypts and villi) and colon (crypts and surface colonocytes). These platforms recapitulate stem cell niche growth factor gradients, allowing for compartmentalization of proliferative and differentiated cells (52, 53).

Another approach to enhance the functionality and applicability of gut-on-a-chip involves the use of scaffold-guided hydrogels that create tube-shaped epithelia with an accessible and perfusable lumen (54). A laser ablation process is used to open a microchannel on the hydrogel, featuring transverse indented pockets. In this system, cells expressing SOX9 (stem cells) and lysozyme (Paneth cells) localize at the base of the pockets, mimicking the intestinal crypts, whereas more differentiated cell types such as absorptive enterocytes, goblet, and enteroendocrine cells are positioned farther from the “crypts,” lining the central channel (54). The model was first described for murine-derived intestinal organoids and then applied to human colon organoids (55). Single-cell RNA-seq revealed that these differentiated colonoids-on-a-chip had higher expression of mature enterocytes compared to undifferentiated conventional 3D colonoids and largely retained the cellular diversity of the native colon.

Further details on organoid-on-a-chip technologies have been described elsewhere (50, 56, 57).

## INCREASING THE COMPLEXITY OF TISSUE CULTURES: INTEGRATION OF HUMAN INTESTINAL ORGANOIDS WITH IMMUNE CELLS

The interaction between intestinal epithelial cells and immune cells is fundamental for maintaining the integrity and function of the gastrointestinal barrier (2). Epithelial cells lining the mucosal surfaces are in constant communication with immune cells through cytokines, chemokines, and extracellular vesicles, as well as by direct contact (3, 58, 59). This dynamic interplay ensures appropriate responses to stimuli, maintaining a balance between effective pathogen clearance and tolerance to food and commensal organisms. Elucidating the molecular mechanisms underlying these interactions is crucial for advancing strategies that can prevent and treat intestinal disorders.

Our group has pioneered the development and characterization of immune enteroids, consisting of intestinal monolayers grown on semipermeable tissue culture inserts and co-cultured with phagocytic cells (Fig. 1C) (10, 11, 60–62). Because these co-cultures recapitulate the co-existence of the intestinal epithelium and immune cells, they offer biological relevance and practicality to study human gut physiology and function. In this system, monocyte-derived macrophages as well as neutrophils are introduced on the basolateral side of the enteroid monolayers (10, 11, 60–62). We have shown that the presence of macrophages enhances mucosal barrier function and that these cells adapt phenotypically in response to epithelial cell signals and apical microbial stimulation (60). In contrast, co-culturing primary neutrophils with enteroid monolayers results in tissue inflammation, neutrophil migration across the epithelial barrier (basolateral-to-apical), and increased intestinal permeability (62). Like macrophages, neutrophils phenotypically adapt within the enteroid microenvironment (62). The enteroid-immune cell co-culture system has proven valuable for modeling dynamic interactions between innate immune cell lineages and epithelial cells, as well as their coordinated responses to environmental stimuli. A challenge that remains is the incorporation of multiple immune cell types simultaneously in a platform that would support cell movement and interactions. Importantly, immune enteroid models have great potential for mechanistic interrogations of host responses by overcoming the limitations of traditional enclosed 3D organoids, which restrict experimental access to the apical surface. Alternatively, reversed-polarity apical-out enteroid models have been described (63, 64). The apical-out configuration preserves epithelial cell differentiation and barrier integrity, enabling the study of pathogens that interact with the apical membrane while maintaining a 3D structure. However, in this model, the basolateral surface is enclosed within the spheroid or partially exposed, limiting its accessibility for experimental manipulation or analysis.

Two recent studies have elegantly reported the generation of human intestinal organoids containing autologous immune cells and responses by tissue-resident lymphocytes (Fig. 1C). Santos et al. described an air-liquid interface (ALI) organoid model produced from duodenal biopsies from both adult individuals with and without celiac disease (13). This new method supports the long-term generation of 3D organoids, which maintain their proliferative capacity while preserving key cellular compartments, including mesenchyme and tissue-resident immune cells (13). In the celiac organoids, gliadin peptides induced epithelial cell apoptosis only when they interacted with human leukocyte antigen HLA-DQ2.5; activation of inflammatory molecules (cytotoxic markers, cytokines, and chemokines) was demonstrated by scRNA-seq (13). Functional experiments revealed that IL-7 produced by organoid mesenchymal cells elicited a CD8^+^ T cell-mediated cytotoxic response that contributed to celiac disease pathogenesis (13). The mechanism underlying the production of IL-7 by mesenchymal cells following gliadin peptide presentation via HLA-DQ2.5 remains unknown. Strikingly, the production of anti-transglutaminase antibodies, a biomarker of celiac disease, is conserved in the ALI organoid model (13). In another study, Recaldin et al. isolated from the same donor’s biopsy both intestinal crypts, for the generation of organoids, and tissue-derived immune cells (12). Immune cells were cryopreserved and introduced into the organoids (12). Most of the isolated immune cells expressed markers of resident memory T cells (T_RM_), and when added to the organoids, they intercalated within the epithelial cells (12). Transcriptome analysis and functional assays of the co-cultured T_RM_ revealed enrichment of gene transcripts associated with cell motility and inflammatory cytokine response, which contributed to epithelial cell damage (12). Both studies reported innovative approaches that open new avenues for investigating tissue-resident cell biology in MHC-matched cell-organoid platforms. These models are ideal to study the induction and deployment of immunity to microbes. However, the difficulty of introducing microbial species into 3D organoids remains one of their limitations.

Together, human immune-organoid co-culture models have revealed mechanisms that regulate barrier function and inflammation and have great potential to decipher fundamental aspects of gut mucosal immunity.

## MODELING DISEASES OF THE HUMAN INTESTINE

### Host-pathogen interactions

Intestinal organoids have been employed as reductionist models to investigate health conditions that impact the human epithelial barrier. They became particularly valuable for studying host interactions with microorganisms that had been previously difficult to investigate due to the lack of human-relevant models. The pathophysiology caused by several invasive and non-invasive enteric bacteria and viruses has been examined extensively in human intestinal organoids; findings have been described in detail elsewhere (65–75). However, only a few studies have interrogated host-pathogen interactions using human intestinal organoid models co-cultured with immune cells. Noel et al. examined responses to enterotoxigenic *Escherichia coli* (ETEC) and enteropathogenic *E. coli* (EPEC) in enteroids co-cultured with macrophages (60). This study showed that subepithelial macrophages were able to sample luminal bacteria by extending membrane projections without disrupting the epithelial barrier (60). These macrophages had adapted and downregulated their production of inflammatory cytokines as compared to uncultured macrophages. A follow-up study confirmed that macrophages co-cultured with crypt- or villus-like epithelium were equally capable of capturing ETEC added to the apical side of the epithelium (61). These observations depict the functional adaptation of macrophages to remove a pathogen while avoiding excessive inflammation.

Our group also reported immune phenotypic adaptation of neutrophils and response to *Shigella flexneri* 2a when co-cultured with ileal enteroid monolayers (62). The presence of *S. flexneri* in the luminal compartment triggered neutrophil migration across the epithelium—a process that, *in vivo*, marks the onset of dysentery—and activated their antimicrobial functions (62). This inflammatory response compromised barrier integrity and facilitated *S. flexneri* invasion, resulting in a double-edged sword effect—while triggering defenses, it enhanced bacterial pathogenesis (62). It is assumed that the same could occur *in vivo*. Importantly, these immune-enteroid co-culture systems revealed changes in immune cell phenotypes that have been overlooked in studies using cancer-derived cell lines or animal models.

A murine 3D bladder epithelial organoid co-cultured with bone marrow-derived neutrophils has been utilized to investigate dynamics of invasion and antimicrobial responses against uropathogenic *E. coli* (UPEC), a major cause of urinary tract infections (76). Spatiotemporal high-resolution microscopy revealed that UPEC evaded neutrophils and antibiotic clearance by rapidly invading deeper epithelial cells within the stratified uroepithelium (76). Although the study was conducted with mouse organoids, it provides insights into a mechanism, previously unrecognized, which might explain the persistent infections with UPEC observed in humans.

Although focused primarily on bacterial infections, these studies have paved the way for the investigation of other enteric pathogens, including viruses and parasites. Research exploring age-dependent immune responses to enteric organisms in humans is needed, particularly to guide health improvements in vulnerable populations.

### Inflammatory bowel disease (IBD)

Crohn’s disease (CD) and ulcerative colitis are types of inflammatory bowel disease that cause chronic inflammation of the digestive tract and severely impair quality of life. Factors contributing to IBD include genetics, an overactive immune system, and microbiome dysbiosis. Organoids have fundamentally contributed to our understanding of these two conditions. Dennison and colleagues identified genetic modifications associated with CD pathogenesis by integrating data from organoid experiments and explant tissue obtained from patients’ mucosal biopsies (77). A reduction of DNA methylation within the NLRC5 promoter—a key transcriptional regulator of the major histocompatibility complex (MHC) class I—increased promoter accessibility, which resulted in elevated NLRC5 mRNA levels, particularly under inflammatory conditions (77). As a corollary, MHC-I expression in intestinal epithelial cells increased T cell activation and inflammation (77). The study illustrates epigenetic changes in stem cells (preserved in the *in vitro* culture) that resulted in inflammation (78, 79).

It has been shown that the sensing of cellular stress, such as extracellular ATP, enhanced TLR5 activation by *E. coli* flagellin (FliC) in human colonoid monolayers, which in turn transformed underlying dendritic cells into pro-inflammatory cells (80). This finding exemplifies how the responses of epithelial cells to stressors (IBD triggers) may result in aberrant stem cell function, leading to altered immune responses. Studies exploring modifications in the stem cell niche and dendritic cell responses in IBD-derived colonoids are warranted to confirm these observations.

Damage to intestinal stem cell function leads to impaired epithelial regeneration, barrier dysfunction, and inflammation, all of which contribute to CD. Leet et al. showed that TNF-α, an effector molecule involved in CD pathogenesis, induced stem cell necroptosis, which disrupted epithelial cell regeneration and reduced the viability of human enteroids (81). Bulk transcriptomic and scRNA-seq analysis revealed a lack of prostaglandin E2 (PGE2) signaling in intestinal stem cells from CD-derived enteroids, whereas exogenous PGE2 restored the proliferative capacity of TNF-α-damaged enteroids, highlighting its potential therapeutic value in intestinal inflammation and epithelial regeneration (81).

Noteworthy, some individuals fail to respond to conventional therapies for IBD (82), underscoring host-phenotypic granularity and the need for personalized treatment. Transcriptional profiles of human colonoids exposed to IBD-associated cytokines (i.e., IFN-γ, TNF-α, IL-13, and IL-17A) revealed both unique and shared signaling pathway activation (83). It was proposed that concurrent activation of cytokine signaling networks may contribute to the lack of response to treatment observed in non-responder patients (83). In fact, gene profiling validation in diseased tissue enabled the classification of IBD patients—both CD and ulcerative colitis—into distinct molecular phenotypes based on a gradient as opposed to discrete cytokine responsiveness (83).

Collectively, these studies highlight the use of human intestinal organoids as powerful translational research tools for the identification of clinical biomarkers and/or novel interventions.

## HUMAN ENTEROID MODEL: ADVANCING PRECISION MEDICINE FROM RESEARCH LAB TO CLINIC

In recent years, enteroid cultures have started to be recognized for their potential to empower personalized medicine. Because enteroids can recapitulate patient-specific features, they are effective tools to identify pathways associated with specific conditions and to facilitate drug discovery (Fig. 1D). As an example, cystic fibrosis (CF) is a genetic disorder caused by mutations in the CFTR gene, which impairs secretion of fluid across the cell membrane and leads to the production of thick and sticky mucus that builds up and causes severe respiratory and digestive issues (84, 85). In the United States, approximately 30,000 individuals are living with CF, and more than 1,700 mutations have been identified (86). Human intestinal organoids have been used to investigate the downstream effects of rare CFTR mutations and response to specific treatment. For instance, Arora et al. examined CFTR function and response to ivacaftor (a CF modulator) through the forskolin (FSK)-induced swelling assay in CF-patients' duodenal enteroid (87). FSK triggers the production of cyclic AMP and activates CFTR channels, which facilitates the secretion of anions and fluid into the enclosed lumen of 3D enteroids (88). This study demonstrated that CFTR restored function through FSK-stimulated fluid transport and confirmed that the response to ivacaftor was specific to this patient’s mutation, as the effect was absent in other CF variants (87). These results identified an effective drug and guided enrollment of the patient in a therapeutic trial. Similarly, other studies reported CF patients’ specific response to drugs in rectal organoids that were associated with clinical outcome (89, 90). Likewise, organoids have been utilized to select adequate therapies for colorectal cancer. Tumor organoid responses from individuals with metastatic gastrointestinal cancer have been shown to replicate patients’ responses observed in the clinic (91), and organoids derived from colorectal cancer patients have been used to predict their responses to chemotherapies (92). Although the number of patients in these studies is limited, these findings support the capability of organoids to inform and predict treatment outcomes. Such a targeted and accurate approach offers several benefits, including minimizing the side effects that accompany cancer therapies. The establishment of a high-throughput organoid system is appealing to streamline drug discovery in a reliable and cost-efficient manner.

Collectively, these results underscore the potential of patient-derived organoids in advancing personalized medicine, facilitating more effective diagnosis and treatment of various conditions.

## CONCLUSIONS

**Fig 2:**
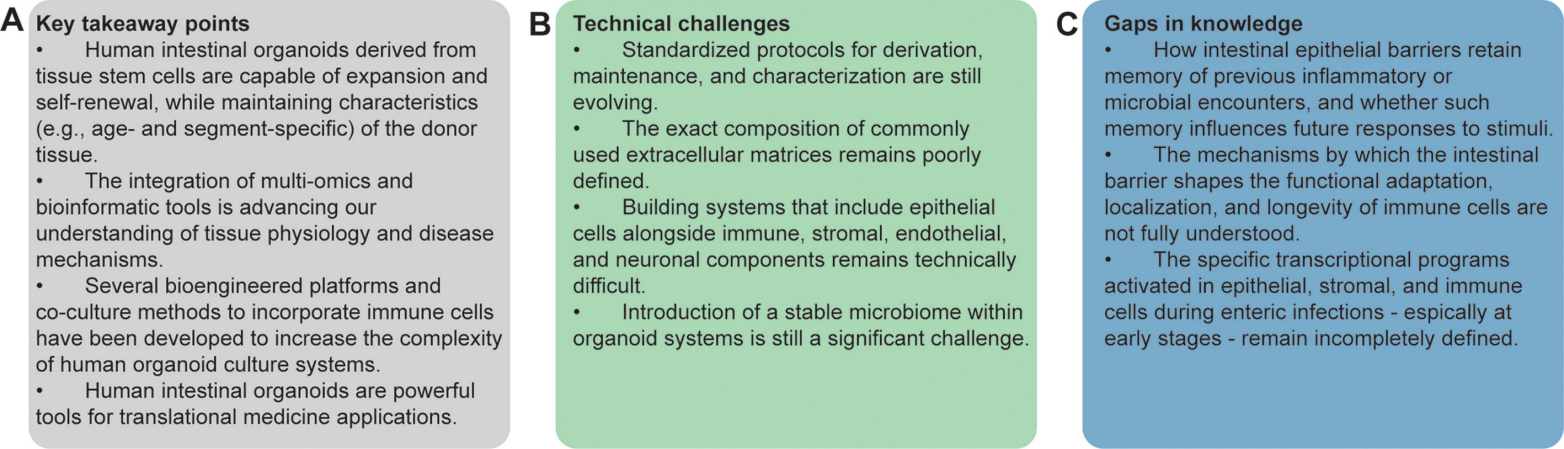
Future directions and outstanding challenges. (**A**) Overview of the principal insights and conclusions presented in this minireview. (**B**) Summary of the key technical hurdles that remain to be addressed in the development and application of intestinal organoid models. (**C**) Unresolved questions and emerging areas of inquiry within the field.

Human intestinal organoid technology has transformed biomedical research by providing physiologically relevant models suitable for studying human intestinal biology and the mechanisms underlying disease (Fig. 2A). Advanced culture systems, derived from tissue stem cells, replicate key aspects of intestinal physiology, including cellular heterogeneity and functional responses to environmental cues. Improvements in media composition through the identification of niche factors and the incorporation of additional components, such as various immune cells, mesenchymal elements, vascular structures, nerve cells, and the microbiota, will enhance the applicability and value of these tissue culture systems to investigate epithelial-immune interactions, host-pathogen responses, and disease pathogenesis (Fig. 2B).

The advent of multi-omics approaches has expanded the potential of organoids to decipher cellular and molecular underpinnings of intestinal homeostasis and disease conditions. In Fig. 2C, I propose a few gaps in knowledge that could be addressed using these integrative technologies. The combination of scRNA-seq, transcriptomic, and proteomic data with organoid cultures is shedding light on complex biological processes, revealing disease pathways, and identifying precise treatment options. These advanced methodologies are driving basic discoveries and empowering translational medicine with unparalleled depth and precision.
